# Bovine FcRn-Mediated Human Immunoglobulin G Transfer across the Milk-Blood Barrier in Transgenic Mice

**DOI:** 10.1371/journal.pone.0115972

**Published:** 2014-12-29

**Authors:** Dan Cui, Linlin Zhang, Jia Li, Yaofeng Zhao, Xiaoxiang Hu, Yunping Dai, Ran Zhang, Ning Li

**Affiliations:** 1 State Key Laboratory for Agrobiotechnology, China Agricultural University, Beijing, China; 2 GenProtein Biotech Ltd., Beijing, China; Xavier Bichat Medical School, INSERM-CNRS - Université Paris Diderot, France

## Abstract

Maternal-fetal IgGs transport occurs either prenatally or postnatally, which confers the newborns with passive immunity before their own immune system has matured. However, little is known about the mechanisms of postnatal IgGs passage in the mammary gland. To investigate how FcRn mediates the IgGs transport in the mammary gland, we first generated bFcRn and anti-HAV mAb transgenic mice, and then obtained HF transgenic mice expressing both transgenes by mating the above two strains. Transgene expression of bFcRn in the four lines was determined by qRT-PCR and western blot. We then localized the expression of bFcRn to the acinar epithelial cells in the mammary gland, and anti-HAV mAb was mainly detected in the acini with weak staining in the acinar epithelial cells. Human IgGs could be detected in both milk and serum of HF transgenic mice by western blot and ELISA. A significantly lower milk to serum ratio of human IgGs in HF mice compared with that of anti-HAV mAb mice, indicating that bFcRn could transport human IgGs across the milk-blood barrier from milk to serum during lactation in HF mice. While, there were no transport of murine IgGs, IgAs, or IgMs. These results provide understandings about the mechanisms of maternal-fetal immunity transfer in the mammary gland.

## Introduction

The passive transfer of Immunoglobulin G (IgG) from mother to offspring is essential for the offspring to gain sufficient immunity and to protect against congenital infection before their immune systems are fully developed [1], [2], which is important for the neonatal during its adaption to the extra-uterine environment. Maternal humoral immunity transfer can occur prenatally and postnatally depending on species and placentations. In rodents, this passage of maternal immunity is mediated by milk after birth [1], [3] and yolk sac antenatally [4], [5]. In ruminants, maternal IgGs are transferred to the offspring exclusively by colostrum in the first several days after parturition [6]. Specifically, there are two critical processes associated with postnatal transfer: IgGs are transferred across the mammary gland into milk and milk digestion by the neonatal intestine. To date, the neonatal Fc neonatal receptor (FcRn) has been well documented to facilitate IgGs transcytosis in the intestine [7], [8], [9], [10]. However, little is known regarding the roles played by FcRn for the IgGs transport in the mammary gland.

FcRn, originally proposed as an IgGs transporter [11], [12], has been characterized as a heterodimer consisting of a 45–53 kDa α-chain that is structurally similar to the major histocompatibility complex (MHC) class I [5], [13] and a 12–14 kDa β2-microglobulin (β2m). This receptor was first identified in rodents that could transfer maternal IgGs from mother to the newborns via the intestine [8], [9], [14]. FcRn is functionally expressed in several tissues and cells [15], [16], [17], [18] that maintain the homeostasis of IgGs [19], [20], [21] and albumin [22], [23], [24] by increasing their half-lives, transfer IgGs across different cells [17], [25], [26], [27], [28], [29], [30], and present antigens [31]. These functions are intimately linked to the receptor’s unique pH-dependent property [32], *i.e.* the ability of FcRn to bind target molecules at an acidic pH (<6.5) and release them at a neutral pH (7.0–7.4) [8], [33], [34].

In ruminants, the transfer of IgGs from blood to milk is believed to occur due to the decrease in plasma IgGs coinciding with its accumulation in milk. And, it is well accepted that IgGs across the mammary barrier to milk is a highly selective process and that only IgGs are transported in large amounts in ruminants [35], [36], [37]. Therefore, these findings suggest that this transfer is most likely a receptor-mediated process. Moreover, FcRn has been detected in the epithelial cells of the mammary gland in different species, including mice [38], brushtail possum [39], bovine [40], pig [41], and sheep [42], in accordance with their different time points of parturition. This indirectly suggests that FcRn expressed in the mammary gland might have the function of regulating IgGs transport from serum to milk. Previous results have demonstrated that FcRn is responsible for IgGs transfer in the intestine, thus we incline to postulate that FcRn probably contribute to the IgGs transfer in ruminant’s mammary gland. The evidence from rodent models, however, supports the possibility that FcRn functions as a recycling, rather than transcytotic transporter of IgGs, due to the reverse correlation of IgGs transport efficiency with the affinity for FcRn in the lactating mice [38], [43]. Thus, how FcRn is involved in the IgGs transport in the mammary gland remains to be elucidated.

To address this question, establishing the proper model to study FcRn-mediated IgGs transcytosis is highly important. Bovine FcRn (bFcRn) has approximately 77% homology with its human counterpart and high affinity with the human IgGs [42], [44]. Therefore, two types of transgenic mouse models were generated (refer to bFcRn mouse and HAV mouse), expressing bFcRn and human anti-HAV mAb specifically in the mammary gland. Mating between the strains produced the bi-transgenic mice (HF mouse), which will be a powerful mouse model to investigate the roles of bFcRn in human IgGs transfer across the milk-blood barrier.

## Materials and Methods

### Transgenic animals

**Figure 1:**
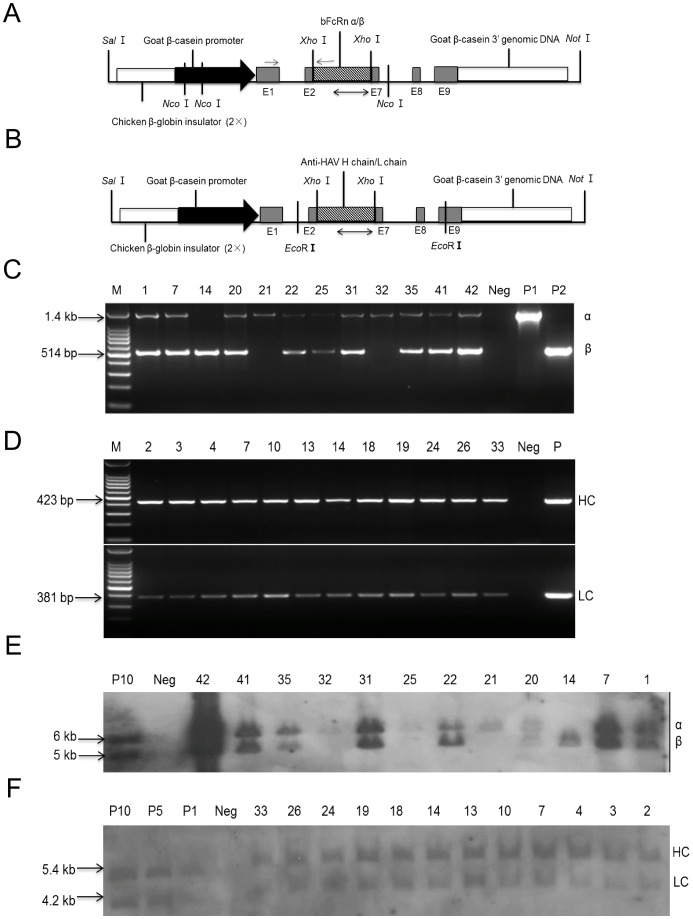
Generation of the bFcRn and anti-HAV mAb transgenic mice. (A–B) Schematic representation of the bFcRn and anti-HAV mAb constructs for microinjection. Chicken β-globin insulator (2x), un-translated exons E1, E8, E9, parts of E2 and E7, and also β-casein 3′ genomic DNA are indicated by bars. Goat β-casein promoter is indicated by solid arrow. The restriction sites of *Sal*I, *Xho*I, *Not*I, *Eco*RI, *Noc*I are shown. The hatched boxes represent insertion of sequences of *bFcRn α, bFcRn β, human IgG HC,* and *human IgG LC* to *Xho*I restriction site, respectively. Double arrows indicate the probes used for the southern blot for *bFcRn* and *anti-HAV mAb* transgenes. Arrows in (A) represent the quantitative real-time PCR primers used for the detection of bFcRn α. (C–D) Identify transgenic founders using PCR. M, 1 kb DNA ladder in (C) or 100 bp DNA ladder in (D); P1, P2, positive plasmids control for *bFcRn α*, *bFcRn β,* respectively; P, positive plasmids control for *human IgG HC* or *human IgG LC*; Neg, genomic DNA from wild-type mice as a negative control. (E–F) Southern blot analysis of transgenes. P10, plasmid control of ten copies of *bFcRn α* or *bFcRn β*; P1, P5, P10, plasmids control equivalents of 1, 5, 10 copies of *human IgG HC* or *human IgG LC*; Neg, genomic DNA from an age matched wild-type mouse as a negative control.

Transgenic mice that specifically express bFcRn and anti-HAV mAb in milk were generated by microinjection. Briefly the bFcRn α-chain and bovine β2-microglobulin (bβ2m) transgenes, containing the pBC1 plasmid backbone of the chicken β-globin insulator, goat β-casein promoter and β-casein 3′ genomic DNA, respectively, were co-microinjected into the pronuclei of fertilized Kunming White eggs to obtain the bFcRn transgenic mice. Similarly, the coding regions of the H chain (HC) and L chain (LC) genes of a human anti-HAV IgG1 mAb with the pBC1 backbone were co-microinjected to obtain the anti-HAV mAb mice (Fig. 1A–B). In this study, we thawed a frozen blastula made by Zhang *et al*. [45] to obtain anti-HAV mAb transgenic mice and ensured that they had an identical expression level of human IgGs. Subsequently, the bFcRn mice and anti-HAV mAb mice were mated with wild-type mice to obtain their offspring. The bFcRn mice were crossed with the anti-HAV mAb mice to obtain the HF transgenic mice, which contained four chains: α-chain, bβ2m, HC and LC. The animal work was approved by the Institutional Animal Care and Use Committee of China Agricultural University (ID: SKLAB-2010-05-01).

### Genotyping of transgenic mice

Genomic DNA was extracted from tail biopsies of mice using phenol method. The presence of the bFcRn transgenes was determined by PCR with the primers pBC-F (5′-GATTGACAAGTAATACGCTGTTTCCTC-3′) and pBC-R (5′-CATCAGAAGTTAAACAGCACAGTTAG-3′). The primers were specific to the β-casein intron 1 and intron 7 sequences and could therefore detect both the α-chain (1.5-kb) and bβ2m (514-bp) genes simultaneously. The thermocycler conditions were: 94°C for 5 min, 30 cycles of 94°C for 30 s, 58°C for 30 s, 72°C for 1 min and 45 s, and 72°C for 7 min. The HC transgene was identified using the primers HC-F (5′-ATGGGTGACAATGACATCCA-3′) and HC-R (5′-ACCTGAGGAGACGGTGACCA-3′); the LC transgene used the LC-F (5′-ATGGGTGACAATGACATCCA-3′) and LC-R (5′-TTTGATCTCGAGCTTGGTCC-3′), which amplified the 423-bp and 381-bp fragments, respectively. The PCR conditions were: 94°C for 5 min, 30 cycles of 94°C for 30 s, 60.5°C for 30 s, 72°C for 45 s, and 72°C for 7 min. Genomic DNA (10 µg) from the bFcRn mice was digested with *Nco* I and analyzed by Southern blotting using the hybridization probes of the 512-bp bFcRn α-chain and 380-bp bβ2m genes labeled with [α-^32^P] dCTP. *Eco*R I restricted genomic DNA from anti-HAV mAb mice was hybridized with the 423-bp HC and 381-bp LC fragments.

### Quantitative real-time PCR

To detect the transcription level of bFcRn, total RNA was isolated from the mammary gland tissues of female transgenic mice from the four transgenic lines during lactation periods using Trizol (Tiangen, China). The primers for bFcRn mRNA expression were Exon1-F (5′-TCCATTCAGCTTCTCCTTCA-3′) and α chain-R (5′- AGCGAGCGATAGTTCTCTGC-3′), which were complementary to the exon 1 of the goat β-casein gene and α-chain gene (We chose a location that had low homology with the endogenous murine FcRn α-chain). Mouse *GAPDH* was used as the internal control and the primers were: GAPDH-F (5′-CGTGCCGCCTGGAGAAACCTG-3′) and GAPDH-F (5′- AGAGTGGGAGTTGCTGTTGAAGTCG-3′). The expression level was analyzed using the 2^−ΔΔct^ method.

DNA from the ear tissues of different bFcRn F0 transgenic mice was also subjected to qRT-PCR for the detection of the copy number. The *Fabpi* gene was used as housekeeping gene for single copy number, and the copy number of transgenic mice was calculated against the standard curve. The primers for bFcRn-α chain were bFcRn-F (5′-GTACCACTTCACCGCCGTGTC-3′) and bFcRn-R (5′-TCAGCCTGCGCCCGTAGATT -3′). The primers for *Fabpi* gene were Fabpi-F (5′- TGTTCAGAGCCAGGAAATCCATA -3′) and Fabpi-R (5′- CATAGGTGTCTCTTTCTTTGGTGTGT -3′). qRT-PCR was performed using SYBR Green (Roche, Switherland) and the LightCycler 480 (Roche, Switherland). The copy number was calculated according to a published protocol [46].

### Preparation of monoclonal antibody (mAbs) against the α-chain epitope of bFcRn

The mouse mAbs were prepared using the custom antibody service provided by Abmart (Shanghai, China). The epitope selected to produce monoclonal antibody had low homology to the murine α-chain to enable endogenous murine FcRn to be distinguished in this study. The specificity of the obtained mAbs was confirmed by western blotting for the α-chain-derived peptide of bFcRn expressed in 293T cells. An anti-bFcRn antibody named K6 was selected for the detection of bFcRn in the western blotting and immunofluorescence assays. The epitope of K6 was specific to AGLAQPLTVE, which was derived from the 279–288 amino acids of the bFcRn α-chain. HRP-conjugated K6 and FITC-conjugated K6 were prepared by CWBiotech (Beijing, China).

### SDS-PAGE and Western blot

Milk and blood from two transgenic lines of mice and non-transgenic mice were collected at day 3 (colostrum) and day 10 (mature milk) of lactation. The milk was diluted with distilled water to 200 µl and defatted by centrifugation at 4°C for 10 min at 4,500×rpm. The serum was separated by centrifugation at 4°C for 5 min at 3,600×rpm and frozen at −80°C for future assay.

The skimmed milk and the serum obtained were mixed with sample buffer and then separated on 10% SDS-PAGE gels under reducing conditions. The proteins were visualized by staining with Coomassie brilliant blue (S4 Fig.). Separated proteins were electrophoretically transferred to nitrocellulose membranes (Amersham Pharmacia, UK), and then incubated overnight in blocking buffer (3% BSA in TBST) at 4°C. The immunodetection of human IgGs was performed with the HRP-conjugated goat anti-human Fab specific antibody (Sigma, USA), and purified human IgG (Sigma, USA) was used as the positive control. Besides, the HRP-conjugated anti-bFcRn mouse antibody (K6) was used for the bFcRn detection in the mammary gland. The images were developed with ECL western blotting reagents (Amersham Biosciences, UK) according to the manufacturer’s instructions. Protein containing the bFcRn was extracted from the mammary gland of cattle at early time of lactation and served as a positive control.

### Determination of Immunoglobulin concentration in milk and serum by ELISA

The amount of recombinant human IgGs, endogenous murine IgGs, IgAs, and IgMs in the colostrum and mature milk from different transgenic groups of mice as well as the non-transgenic mice was determined using the enzyme-linked immunosorbent assay (ELISA) kit for human IgG, murine IgG, IgA, and IgM (Bethyl, Montgomery, TX, USA), respectively, according to the manufacturer’s instructions. The absorbance of the products was measured at 450 nm using a model 550 microplate reader (Bio-Rad, Hercules, CA).

### Immunofluorescent staining

Immunofluorescence was performed as described previously [47]. Mice at mid-lactation were euthanatized by cervical dislocation and mammary glands were isolated immediately from underneath the skins with blades. Mammary gland tissues from mid-lactation were fixed in 4% PFA, paraffin-embedded and cut into 5-µm sections. For immunofluorescence staining, antigen-retrieval was performed by heating slides to 95°C for 10 min in 0.01 M citrate buffer (pH 6.0) in a microwave oven. Paraffin sections were pretreated, incubated with antibodies and counterstained with DAPI in mounting medium. Human IgG was detected by the Cy3-conjugated goat anti-human IgG antibody (CWBiotech, China). The FITC-conjugated anti-bFcRn mouse mAb (K6) was used for the detection of bFcRn. Immunofluorescent staining was observed under Olympus BX50 Fluorescence Microscope (Olympus, Japan).

### Statistical analysis

The experimental data were analyzed by a one-way ANOVA and Student’s test using SPSS15.0 software. A *P* value <0.05 was considered significant.

## Results

### Generation and characterization of bFcRn and anti-HAV mAb transgenic mice

To obtain the bFcRn mice, the exiting cassettes expressing the bFcRn α-chain and bβ2m genes were co-injected into the fertilized mouse eggs. Twelve bFcRn transgenic founders (three females numbered 31, 41, 42 and the rest were nine male lines, among them line-14, -21, -32 harbored either α-chain or β-chain of bFcRn) were produced, which were first identified by PCR (Fig. 1C) and then confirmed by southern blot analysis (Fig. 1E). Four lines (lines-1, -7, -20, -42) in bFcRn transgenic mice were chose for subsequent experiments. The transgenes copy numbers of the bFcRn transgenic mice in those lines were determined by quantitative real-time PCR, which showed that lines-1 (3 copies), -7 (3 copies), -20 (1 copies), -42 (9 copies), ranging from one to nine. The mice expressing human anti-HAV IgG1 used in this study originated from one frozen reservation embryo that had the highest expression level of 17.6 mg/mL mAbs detected in milk previously, which ensured that the expression level of the mAbs was almost the same in each anti-HAV transgenic mouse. The transgenes integration of human anti-HAV IgG1 was confirmed by PCR (Fig. 1D) and southern blot (Fig. 1F). Transgenic founders in both types of transgenic mice were mated with wild-type mice and all of them could transmit the transgenes to their offspring (data not shown). We selected offspring from the two types of transgenic mice to obtain enough HF transgenic mice that contained the bFcRn α, β subunits and the anti-HAV mAb HC, LC by mating in each of the four lines.

### The bFcRn was expressed in the mammary gland of transgenic mice

**Figure 2:**
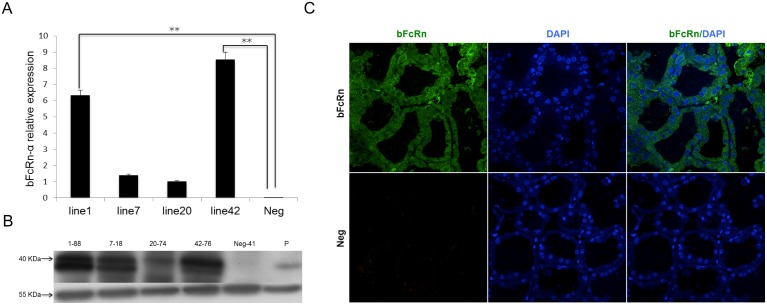
Mammary gland expression of the bFcRn transgenes in transgenic mice. (A) *bFcRn α* mRNA levels in the mammary gland of transgenic mice. The expression levels were determined by the expression related to *GAPDH* (an internal control). The data were combined from three independent experiments; the bars represent the means ± SD (n = 7–8 mice per group); Neg, wild-type mice as a control; **P<0.01. (B) Western blot of bFcRn in the four lines of transgenic mice. HRP-conjugated K6 antibody against a polypeptide (AGLAQPLTVE) representing amino acids 279–288 was used. Tubulin was used as a loading control. Neg-41, protein from a wild-type mouse as a control; P, bovine mammary gland. (C) Immunofluorescent analysis of the mammary gland during lactation. Tissue sections prepared from the mammary gland were stained with the FITC-conjugated anti-bFcRn α antibody (K6) (Green). DAPI (blue) indicated nuclear staining. The data are representative of at least three sections. Images were obtained with a 40X water objective lens.

**Figure 3:**
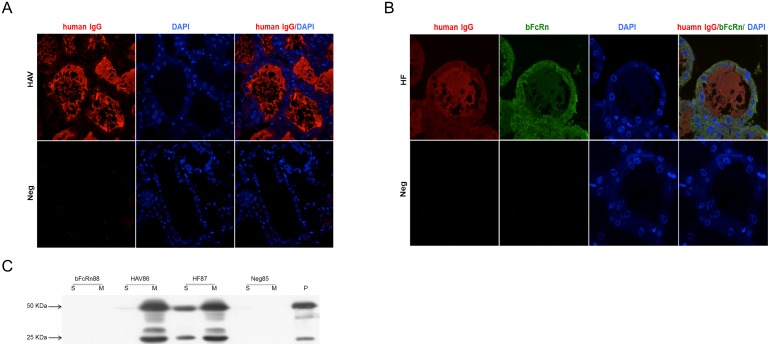
Analysis of the distribution of human IgGs in anti-HAV mAb and HF transgenic mice. Human IgGs distribution in the anti-HAV mAb transgenic mice (A) and co-localization of bFcRn and human IgGs in the HF transgenic mice (B). Tissue sections prepared from the mammary gland of anti-HAV mAb mice were incubated with Cy3-conjugated (red) goat anti-human IgG antibody. For the HF mice, the sections were hybridized with a combination of Cy3-conjugated (red) goat anti-human IgG antibody and FITC-conjugated anti-bFcRn α antibody (K6). The nucleus was stained with DAPI (blue). The data obtained are representative of at least three sections. Images were obtained with a 40X water objective lens in (A) and a 100X oil objective lens in (B). (C) Human IgGs were transferred from milk to serum in the HF transgenic mice. Milk and serum (day3) were performed western blot using goat anti-human IgG antibody in four different genotypes of mice from line-1. S, serum; M, milk; P, positive control, human IgG.

To determine the relative levels of bFcRn expression in the four founder lines, we performed quantitative real-time PCR using RNA extracted from the mammary gland of the transgenic female mice at mid-lactation (day 10). Our results showed (Fig. 2A) that line-42 had the highest α subunit expression compared with the other three lines. Besides, the expression levels of line-1 and line-42 were significantly higher than those of wild-type mice (*p*<0.01), whereas line-7 and line -20 had lower levels of bFcRn gene expression. Thus, we selected line-1 and line-42 for further analysis. These results were consistent with RT-PCR assay performed for bFcRn α subunit (S1 Fig.). To further detect the bFcRn protein in each line, proteins obtained from the mammary glands during lactation were subjected to western blotting using K6 mAb that was validated *in vitro* (S2 Fig.). As shown in (Fig. 2B), a strong band was detected for the α-chain with the expected molecular weight (MW) of 40 kDa in all four lines. Weak reactivity was shown in transgenic line-7 and line-20 compared with that of line-1 and line-42, which was in agreement with the RNA levels in the four lines. No bFcRn could be detected in wild-type mice. Subsequently, to further determine the localization of bFcRn, sections of mammary gland from line-1 were incubated with the anti-bFcRn mouse mAb using an immunofluorescence assay. Marked staining was observed in the epithelial cells of the acini in both of bFcRn (Fig. 2C) and HF (Fig. 3B) transgenic mice, which was diffuse in the epithelial cells but concentrated at the apical sites. In contrast, the mammary gland sections of wild-type mice showed no staining.

### Distribution of human IgGs in HF and anti-HAV transgenic mice

Previous reports has shown that IgGs is transported by FcRn in other barrier systems, so we hypothesized that bFcRn specifically expressed in the mammary gland could affect the distribution of human IgGs in HF mice. Firstly, anti-HAV mAb localization was determined both in anti-HAV mAb (Fig. 3A and S3 Fig.) and HF (Fig. 3B and S3 Fig.) transgenic mice. We observed strong staining in the acini of both HF and anti-HAV mAb transgenic mice, weak staining in acinar epithelial cells of HF mice, and even less staining in acinar epithelial cells of anti-HAV mAb mice. We then examined the anti-HAV IgGs levels by SDS-PAGE (S4 Fig.) and western blot in serum and milk of HF, bFcRn, anti-HAV mAb transgenic mice and wild type mice (the latter two types of transgenic mice were used as controls) in both lines. IgGs could be detected both in the colostrum and serum of HF mice, whereas it was only observed in the colostrum of anti-HAV mAb transgenic mice, as shown in (Fig. 3C and S5B Fig.). And the same results were obtained when it came to the mid-lactation milk and serum (S5A and S5C Fig.). No human IgGs were detected in bFcRn transgenic mice and wild-type ones. Moreover, after the uptake of human IgGs by the neonatal in the intestine, the molecular weight of human IgG LC is larger than that in anti-HAV mAb transgenic mice (S6 Fig.). These results indicated that human IgGs could be efficiently expressed in the mammary gland of transgenic mice and the expression of bFcRn could change the distribution of human IgGs in HF mice compared with anti-HAV mAb mice.

### Functional analysis of bFcRn in the mammary gland of transgenic mice

**Figure 4:**
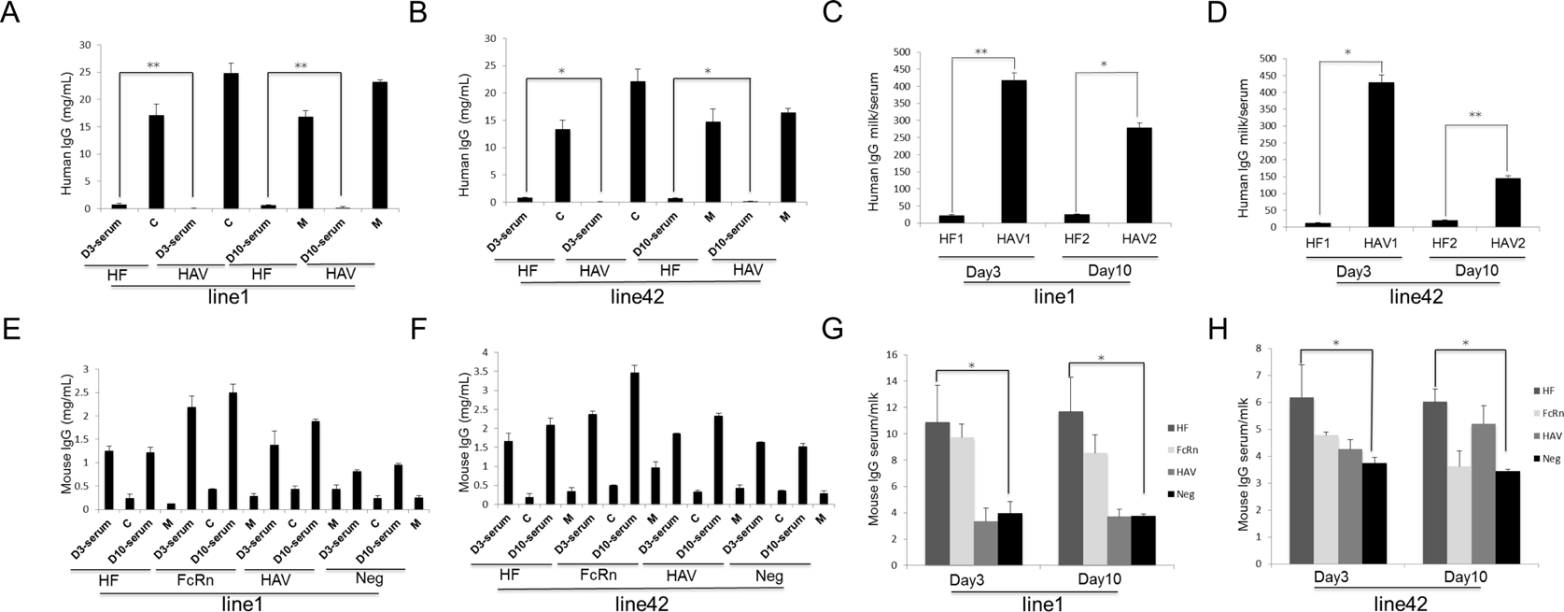
ELISA analysis of human IgGs in the mammary gland of transgenic mice. Human IgGs were detected in the serum of HF mice (A–B) and the milk/serum ratio was significant lower in HF transgenic mice (C–D). The milk/serum ratio is used as an index for the transfer of human IgGs from milk to serum in the mammary gland. The concentrations of murine IgGs (E–F) and the ratio of serum/milk (G–H) indicated that there was no transfer of murine IgGs by bFcRn. The ratio of serum/milk is used as an index for the transfer of murine IgGs from serum to milk. D3-serum, serum obtained on day 3 of lactation; C, colostrum collected on day 3 of lactation; D10-serum, serum obtained on day 10 of lactation; M, milk collected on day 10 of lactation. The data were combined from three independent experiments; the bars represent the means ± SD (n = 7–8 mice per group); *P<0.05; **P<0.01.

Western blot showed that human IgGs were detected in the serum of HF mice, suggesting the transport of human IgGs by bFcRn in the HF mice. We then measured the amounts of human IgGs transported by bFcRn in HF transgenic mice. The skimmed milk both at day 3, day 10 and the corresponding serum in line-1 and line-42 were measured using ELISA. The results were consistent with western blotting. Human IgGs could be detected in the milk of both the anti-HAV mAb and HF groups and that human IgGs levels in the serum of the HF group were significantly higher than that in the anti-HAV mAb group (*p*<0.05) (Fig. 4A–B). Next, the milk/serum ratio of human IgGs was used as an index to detect human IgGs transport, and the ratio was significantly lower in the HF group than in the anti-HAV mAb group during both the colostrum and mature milk periods in both lines (*p*<0.05) (Fig. 4C–D). Taken together, these data strongly support the hypothesis that the mammary gland specifically expressed bFcRn could transport human IgGs from milk to serum.

To further investigate whether the FcRn has the function of transporting endogenous IgGs, murine IgGs levels in the milk and serum of line-1 and line-42 in three transgenic groups at day 3 and day 10 were measured using ELISA, with wild-type murine milk and serum as controls. The bFcRn transgenic mice had higher concentrations of murine IgGs than wild-type mice in both milk and serum (line-1 serum: *p* = 0.021 at day 3, *p* = 0.000 at day 10; line-1 milk: *p* = 0.042 at day 3, *p* = 0.047 at day 10; line-42 serum: *p* = 0.040 at day 3, *p* = 0.003 at day 10; line-42 milk: *p* = 0.032 at day 3, *p* = 0.003 at day 10;) (Fig. 4E–F). The HF mice had higher levels of murine IgGs than wild-type mice in serum (line-1: *p* = 0.034 at day 3, *p* = 0.011 at day 10; line-42: *p* = 0.040 at day 3, *p* = 0.010 at day 10), but the level of murine IgGs were lower than that in the bFcRn transgenic mice (Fig. 4E–F). Moreover, the serum/milk ratio of the murine IgGs isotype was used as an index for endogenous IgGs transport. There was no significant difference between the bFcRn transgenic mice and wild-type mice, indicating there was no bFcRn-mediated murine IgGs transport from serum to milk (Fig. 4G–H). Interestingly, the anti-HAV mAb transgenic mice also had an elevated level of endogenous IgGs compared with wild-type mice. Besides, HF mice had significantly higher ratio of serum/milk than that of the wild-type mice (*p*<0.05).

### bFcRn could not transport other immunoglobulin subclasses across mammary gland

**Figure 5:**
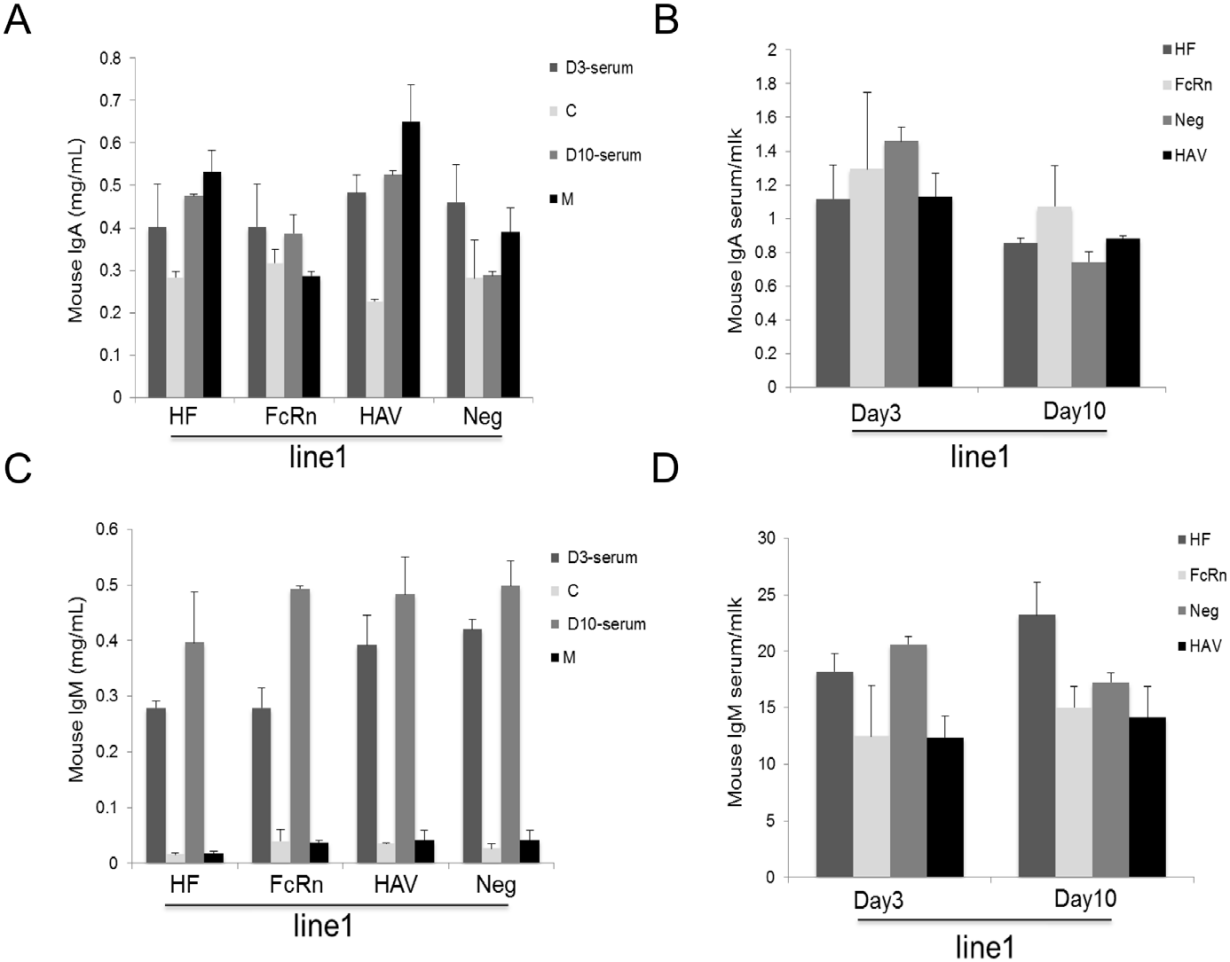
bFcRn cannot transport murine IgAs and IgMs across the mammary gland. bFcRn could neither increase the concentration of IgAs and IgMs (A and C) nor transport them across the milk-blood barrier in the mammary gland (B and D). The ratio of serum/milk indicates the transfer of IgAs or IgMs from serum to milk. The data were combined from three independent experiments; the bars represent the means ± SD (n = 7–8 mice per group).

The above data suggested that bFcRn could transport human IgGs from milk to serum but did not facilitate endogenous IgGs across the mammary gland. To investigate whether the effect of bFcRn could extend to other murine immunoglobulin subclasses, the levels of IgAs and IgMs in the milk and serum of the three transgenic groups in line-1 were measured by ELISA. No significant differences in the transport of IgAs and IgMs were observed with respect to the concentrations (Fig. 5A and 5C) and the serum/milk ratios (Fig. 5B and 5D) in each group. These data suggested that bFcRn could directly transport human IgGs in the mammary gland from milk to serum. In terms of the other immunoglobulin subclasses, bFcRn was devoid of this transportation effect, most likely due to a lack of specific binding between bFcRn and these immunoglobulins.

## Discussion

In our study, we produced HF transgenic mice that specifically expressed bFcRn and human IgGs in the mammary gland. Our results showed that bFcRn could transport a significantly greater amount of human IgGs from milk to serum compared with anti-HAV mAb mice, but that no transfer of mouse endogenous IgsGs, IgAs, and IgMs. These findings provide understanding of how human immunoglobulins are transported by bFcRn in the mammary gland during passive maternal-fetal immunity transfer.

For maternal-fetal passive immunity transfer, immunoglobulins have to cross both the mammary gland and the neonatal intestine barrier [22]. However, the tight junctions that formed during lactation make it impossible for IgGs to cross the mammary gland through intracellular junctions. Moreover, immunoglobulins, recognized as macromolecules, are not able to traverse epithelial barriers intact by passive diffusion [48], [49]. Therefore, this paracellular pathway of IgGs is most often receptor-mediated. For example, FcRn binds to IgGs in cells and a variety of barriers to aid IgGs transfer across different cells [26], [50], [51], [52]. In our study, we generated a mouse model that expressed bFcRn and human IgGs in a mammary-gland-specific manner. This could specifically localize the human IgGs and bFcRn to the mammary gland instead of systemic expression, which excluded any interference of human IgGs from other origins. The anti-HAV mAb transgenic mice were generated from a blastula that could guarantee the same expression level of human IgGs in this study. In addition, bFcRn binds human IgGs much more efficiently than bovine IgGs or murine IgGs [44], [53], providing a sensitive measurement of the dynamic distribution of human IgGs during immunity transfer at the blood-milk barrier. This is supposed to be an effective model for investigating maternal-fetal immunity transfer due to the limitations and ethical considerations surrounding human research.

Our results showed that bFcRn were expressed in the epithelial cells of the acini in both the bFcRn and HF transgenic mice. This expression pattern of bFcRn in transgenic mice is in agreement with the expression pattern of the mouse endogenous FcRn [38]. Human IgGs were detected mostly in the acini with weak staining in acinar epithelial cells in HF mice, whereas they showed a strong staining in the acini and much less weaker staining in the acinar epithelial cells in the anti-HAV mAb transgenic mice. This finding suggested that anti-HAV mAb was expressed in the epithelial cells of acini and then were secreted into the acini. However, bFcRn has to bind human IgGs first during the process of mammary-blood transfer. We thus questioned whether bFcRn binds to human IgGs by taking them up from milk after its secretion or binds to human IgGs in the epithelial cell before its secretion. Interestingly, our results favorably agreed with the former hypothesis, showing that the co-localization of human IgGs and bFcRn in the acinar epithelial cells of HF transgenic mice with much less staining of human IgGs in the acinar epithelial cells of anti-HAV transgenic mice. We cannot exclude, however, that there was partial binding to human IgGs in the epithelial cells of the acini before secretion.

The over-expression of bFcRn and human IgGs in mouse mammary gland resulted in bFcRn-medicated human IgGs transferred from milk to serum. This result leads us to predict the process of this transfer. A pH-dependent interaction has been well documented to be necessary for the binding of IgGs by FcRn [9], [33], [54] arising from the titration of histidine residues [32], [33]. In the steady state, most cells are bathed in a neutral pH together with weak expression of FcRn on the cell surface [4], [16], [55], leading to the rare take-up of IgGs by FcRn at extracellular compartments. Therefore, human IgGs were most likely pinocytosed nonspecifically by fluid-phase endocytosis and delivered to sorting endosomes, bound specifically to FcRn in acidic endosomes (pH<6.5) [56], subsequently transcytosed to the basolateral surface of the epithelial cells of acini and then released at neutral pH (7.0–7.4). Excess human IgGs could not bind FcRn were destined for degradation by trafficking to lysosomes. This prediction is in line with the proposed mechanism for IgGs transfer in rat yolk sac [4], [5] and human placental syncytiotrophoblast [57], [58] where an acid pH is not a prerequisite for receptor-mediated cell surface binding for IgGs uptake [56]. However, it is in contrast with the transfer at neonatal intestine [3], [8], [9], [59] where the acid pH allows IgGs take-up at the cell surface.

Our results suggested that human IgGs transport by bFcRn from milk to serum was an apical to basolateral transfer in the mammary gland of lactating mice, which is also in accordance with the data obtained in hepatocyte [15], [60]. FcRn was originally defined as a transporting receptor for the absorption of IgG across the intestine in suckling rodents [9] and was down regulated sharply after weaning [12], [14]. This process is an apical to basolateral transfer of maternal IgGs in the intestinal epithelial cells. With the detection of other cell types *in vitro*, FcRn functions as a bidirectional transcytosis [25], [28], [29], [30], [61], [62], [63] receptor of IgGs in polarized epithelial cells despite its property of a favorable direction in different cell types. In transfected polarized cells, human FcRn has been reported to localize at basolateral sites, facilitating a basolateral-to-apical transport [50], whereas rat FcRn is preferentially distributed at apical sites, resulting in an apical-to-basolateral transfer [26]. This difference has been attributed to the different potential glycosylation sites in FcRn [64], [52], *i.e.* the rat has four sites and human has only one such site. Differential sorting of FcRn has also been reported, which results from the different sorting target motifs in the cytoplasmic domain [65]. bFcRn has one potential glycosylation site and a cytoplasmic tail that is ten amino acids shorter [40] than its rodent counterpart, which can still facilitate human IgGs transfer from apical to basolateral in the mammary gland of lactating mice. This finding again suggests that there may be fundamental differences in regulating FcRn-mediated-IgG transcytosis among different cell types. To understand the dynamic process of this transcytosis, further studies need to be performed using mammary gland epithelial cells of HF transgenic mice.

Although we detected a significant transfer of human IgGs from milk to serum, no transfer of murine IgGs were observed in either direction. Our results showed that the ratio of serum/milk of murine IgGs in HF mice was significantly higher than that in wild-type mice. However, we could not confirm that bFcRn was able to transport murine IgGs from serum to milk because we could not eliminate the effects of human IgGs on murine IgGs’ distribution and metabolism in HF mice. Meanwhile, there was no significant difference between the bFcRn and wild-type mice with regard to the murine IgGs serum/milk ratio. Our data, therefore, could not support the bFcRn-mediated transport of murine IgGs from serum to milk, and instead, murine IgGs may be recycled back into serum. This possibility corroborates the data reported by Cianga *et al*. [38] and Lu *et al*. [43], who suggested a reverse correlation between IgGs affinity and the efficiency of transcytosis. FcRn-mediated recycling or transcytosis of IgsGs is known to depend on the cell types, FcRn expression levels, ligand affinity, and the physiological conditions [55]. Furthermore, bFcRn can both bind human and murine IgGs, albeit with a greater affinity to human IgGs than murine IgGs [44], [53]. This competition between the two types of IgGs might also be an explanation for the lack of murine IgGs transfer from serum to milk.

bFcRn could neither increase the concentration of murine IgAs, IgMs nor transport them across milk-blood barrier in this study. This observation is in line with previous results demonstrated by Lu *et al*. [43], which probably resulted from the fact that bFcRn cannot specifically bind to IgAs and IgMs to increase their concentrations. However, FcRn can bind IgGs to avoid degradation and thus extend its half-life; and bFcRn improved the concentration of murine IgGs in both serum and milk of bFcRn transgenic mice in this study. In terms of the concentration of murine IgGs in HF mice, we only observed significantly higher concentration of murine IgGs in the serum compared with wild-type mice. The different concentrations of murine IgGs between bFcRn and HF transgenic mice may attribute to the changes of murine IgGs’ metabolism or distribution caused by over-expression of human IgGs in HF mice. Further study needs to be conducted to address this question.

In summary, we generated transgenic mice with mammary-specific expression of bFcRn and human anti-HAV mAb to demonstrate how human IgGs were transferred by bFcRn across the blood-mammary barrier. The locations of bFcRn and human IgGs have been investigated by immunofluorescence, showing that bFcRn was localized in the acinar epithelial cell and human IgGs was expressed in the acinar epithelial cells and then secreted into the acini. The bFcRn specifically expressed in mice could facilitate the transport of human IgGs from milk to serum at the milk-blood barrier during lactation. These data sheds light on the mechanisms of maternal-fetal immunity transfer in the mammary gland.

## Supporting Information

S1 Fig **RT-PCR analysis of bFcRn α-chain mRNA level in the bFcRn transgenic mice.** 1–8, 7–18, 20–60, 42–75, bFcRn transgenic mice from the four founder lines (lines-1, -7, -20, -42). β actin is used as an internal control. M, 100 bp DNA ladder; Neg, mRNA from wild-type mice. The data represent three independent experiments.
(TIF)

S2 Fig **Validation of bFcRn mAbs using FcRn protein overexpressed in 293T cells.** bFcRn with myc tag (S2A) or bFcRn with EGFP tag (S2B) overexpressed in 293T cells were used to validate bFcRn mAbs. F16, L10, K6, C1, 10, the names of bFcRn mAbs; Neg, Neg1, Neg2, protein obtained from 293T cells untransfected with vectors bFcRn-myc, bFcRn-EGFP, murine FcRn-EGFP, respectively; k6, K6 mAb hybridized with proteins obtained from 293T cells transfected with murine FcRn- EGFP, indicating that K6 mAb could not cross react with murine FcRn; 48 kDa, 68 kDa, the molecular weight of recombinant bFcRn protein with myc or EGFP tag, respectively.
(TIF)

S3 Fig **Distribution of human IgGs in the anti-HAV mAb and HF transgenic mice by immunochemical staining.** The data obtained are representative of at least three sections. Scale bar, 50 µm.
(TIF)

S4 Fig **SDS-PAGE of human IgGs in different types of transgenic mice and wild-type mice in line-1.** S, serum; M, milk; P, human IgG.
(TIF)

S5 Fig **Human IgGs were transferred from milk to serum in the HF transgenic mice.** Milk and serum (day 10) in line 1 (S5A) and both day 3 (S5B) and day10 (S5C) in line 42 were performed western blot using goat anti-human IgG antibody in four different genotypes of mice. S, serum; M, milk; P, human IgG.
(TIF)

S6 Fig **Maternal-fetal human IgGs transfer through suckling.** Day3, Day10, two time points were chose to collect milk and serum samples; S, M, s, maternal serum, maternal milk, serum of the neonatal. After the uptake of human IgGs by the neonatal in the intestine, the molecular weight of human IgG LC is larger than that in anti-HAV mAb transgenic mice.
(TIF)
