# Supplementary figures and images for: Bovine FcRn-Mediated Human Immunoglobulin G Transfer across the Milk-Blood Barrier in Transgenic Mice
Source: PLoS One. 2014 Dec 29;9(12):e115972. doi: 10.1371/journal.pone.0115972 (PMC4278800; doi:10.1371/journal.pone.0115972)

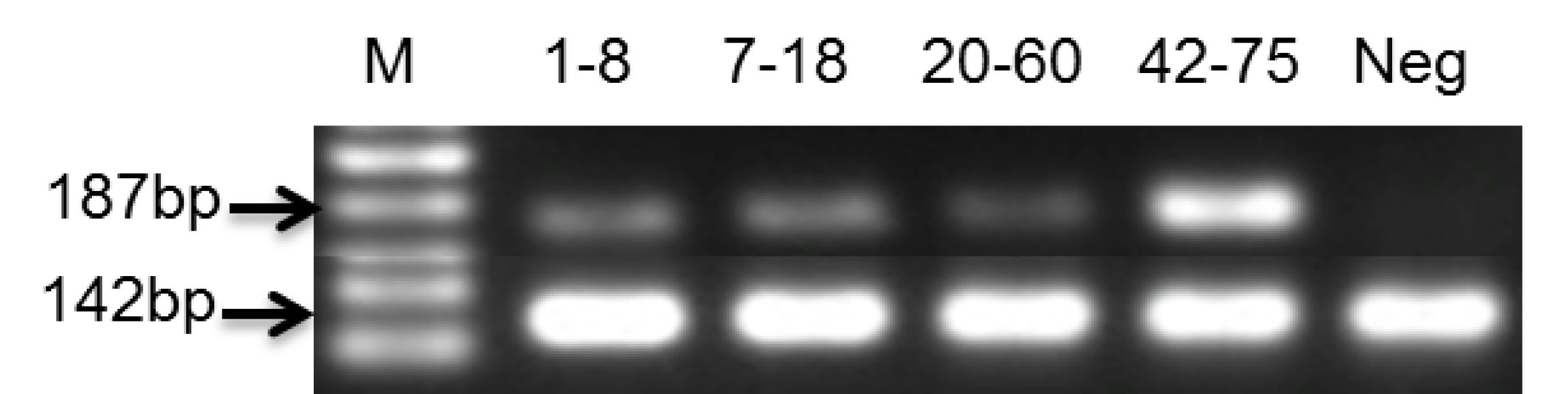

Supplement: S1 Fig — RT-PCR analysis of bFcRn α-chain mRNA level in the bFcRn transgenic mice. 1–8, 7–18, 20–60, 42–75, bFcRn transgenic mice from the four founder lines (lines-1, -7, -20, -42). β actin is used as an internal control. M, 100 bp DNA ladder; Neg, mRNA from wild-type mice. The data represent three independent experiments. (TIF) [file pone.0115972.s001.tif]

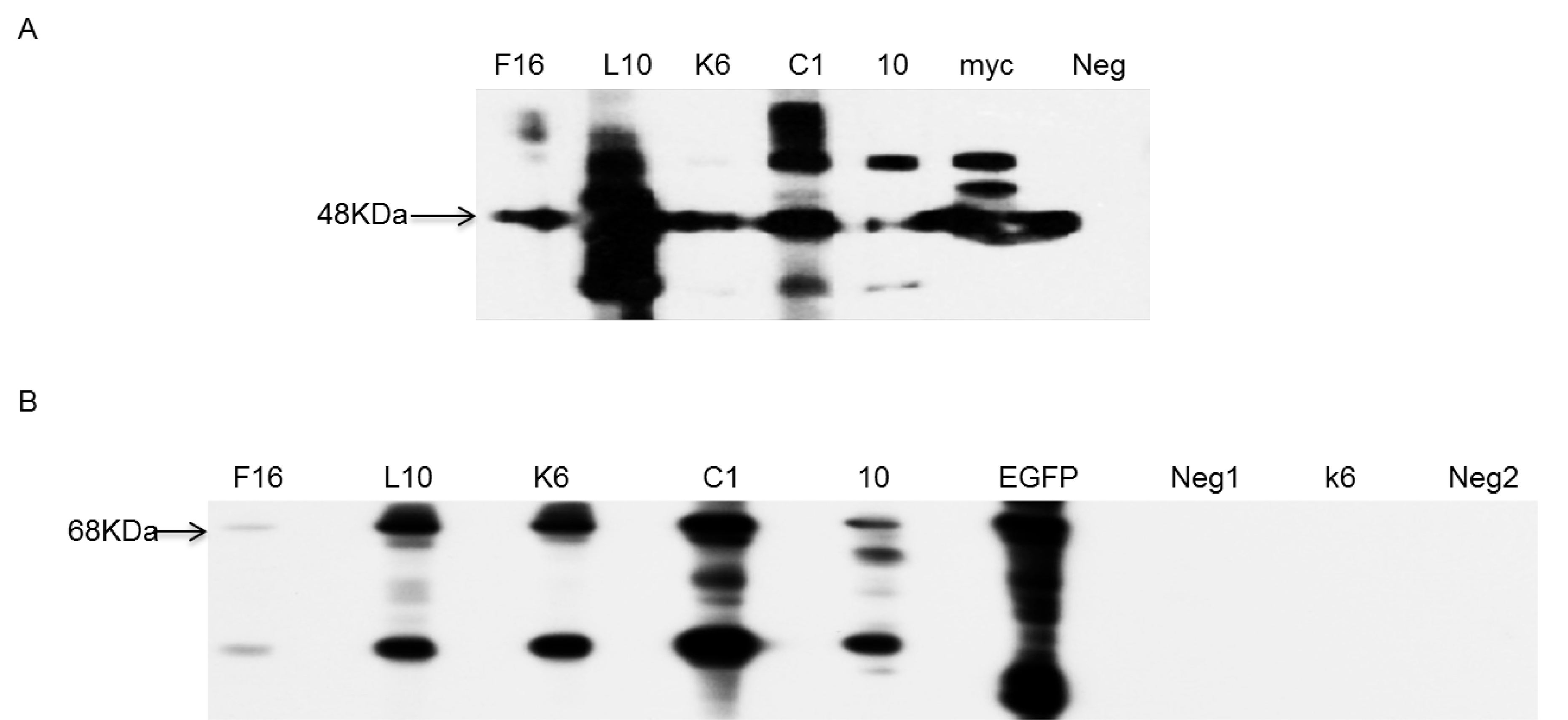

Supplement: S2 Fig — Validation of bFcRn mAbs using FcRn protein overexpressed in 293T cells. bFcRn with myc tag (S2A) or bFcRn with EGFP tag (S2B) overexpressed in 293T cells were used to validate bFcRn mAbs. F16, L10, K6, C1, 10, the names of bFcRn mAbs; Neg, Neg1, Neg2, protein obtained from 293T cells untransfected with vectors bFcRn-myc, bFcRn-EGFP, murine FcRn-EGFP, respectively; k6, K6 mAb hybridized with proteins obtained from 293T cells transfected with murine FcRn- EGFP, indicating that K6 mAb could not cross react with murine FcRn; 48 kDa, 68 kDa, the molecular weight of recombinant bFcRn protein with myc or EGFP tag, respectively. (TIF) [file pone.0115972.s002.tif]

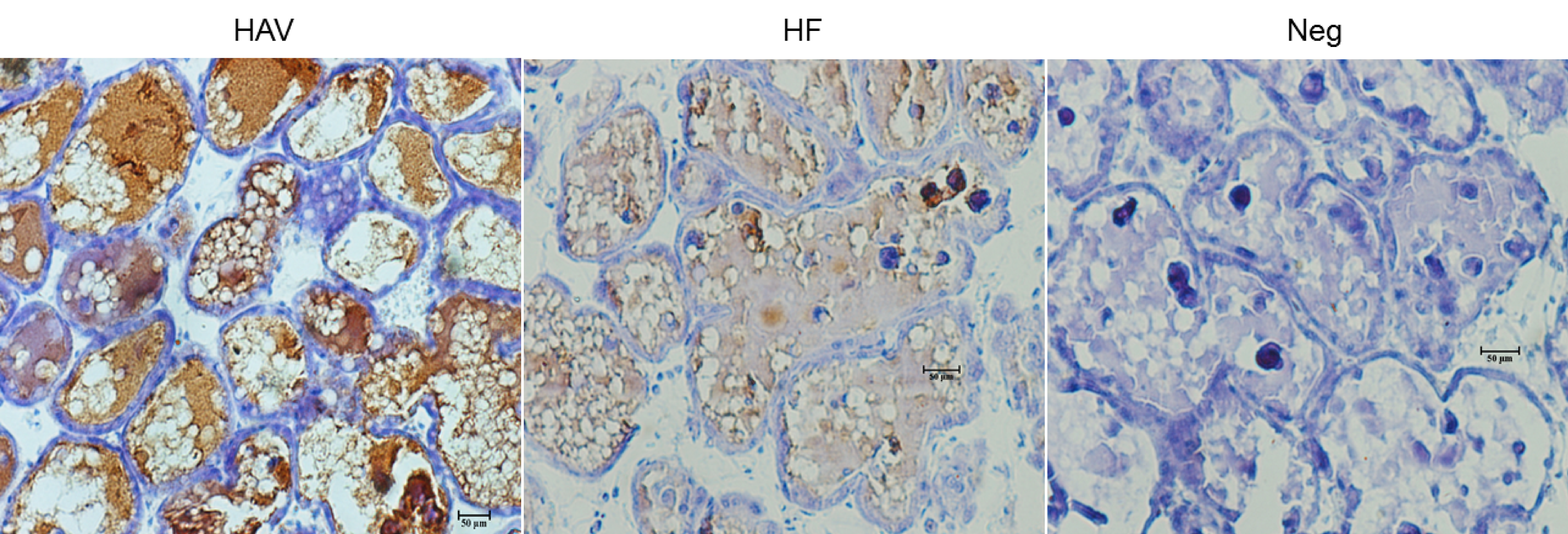

Supplement: S3 Fig — Distribution of human IgGs in the anti-HAV mAb and HF transgenic mice by immunochemical staining. The data obtained are representative of at least three sections. Scale bar, 50 µm. (TIF) [file pone.0115972.s003.tif]

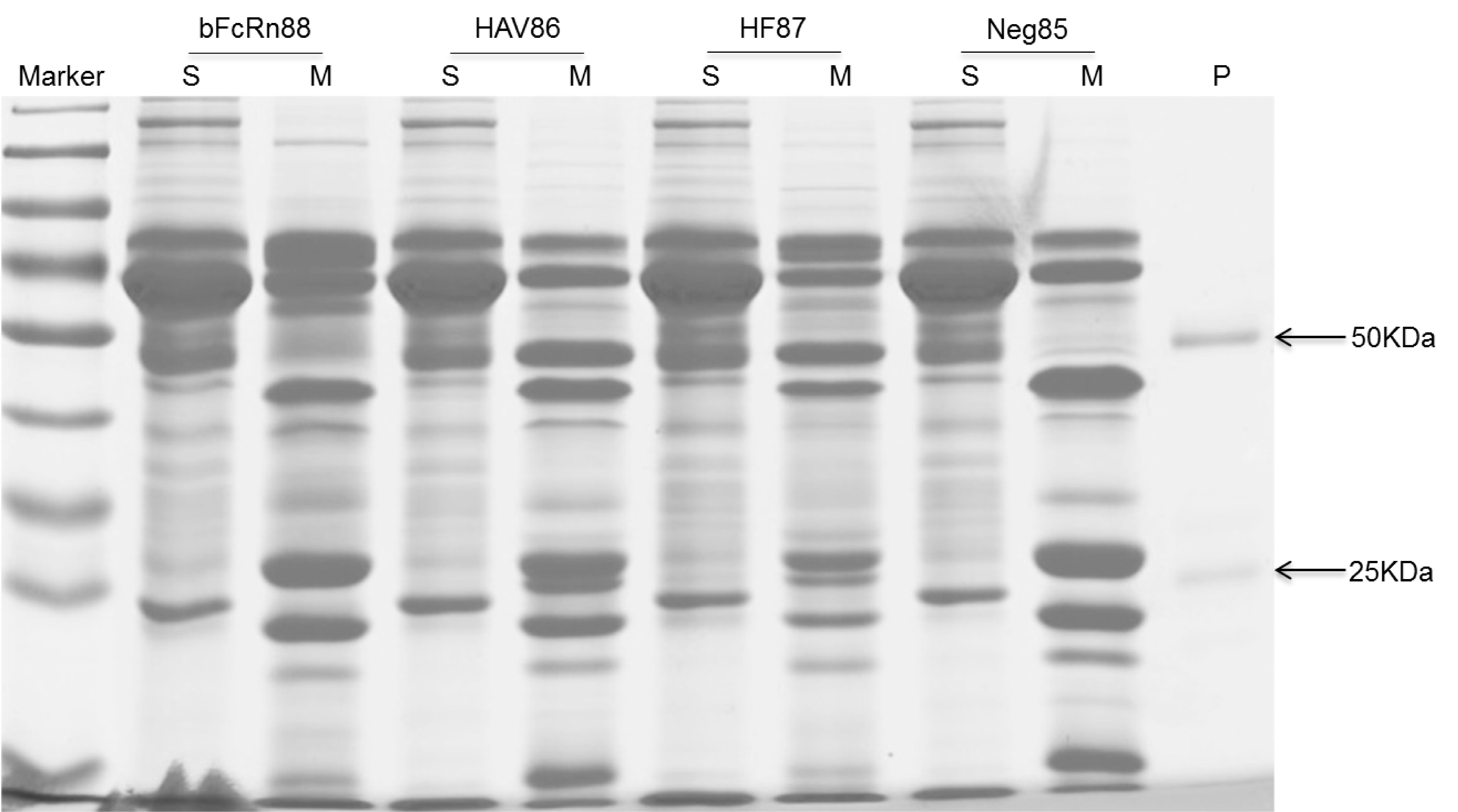

Supplement: S4 Fig — SDS-PAGE of human IgGs in different types of transgenic mice and wild-type mice in line-1. S, serum; M, milk; P, human IgG. (TIF) [file pone.0115972.s004.tif]

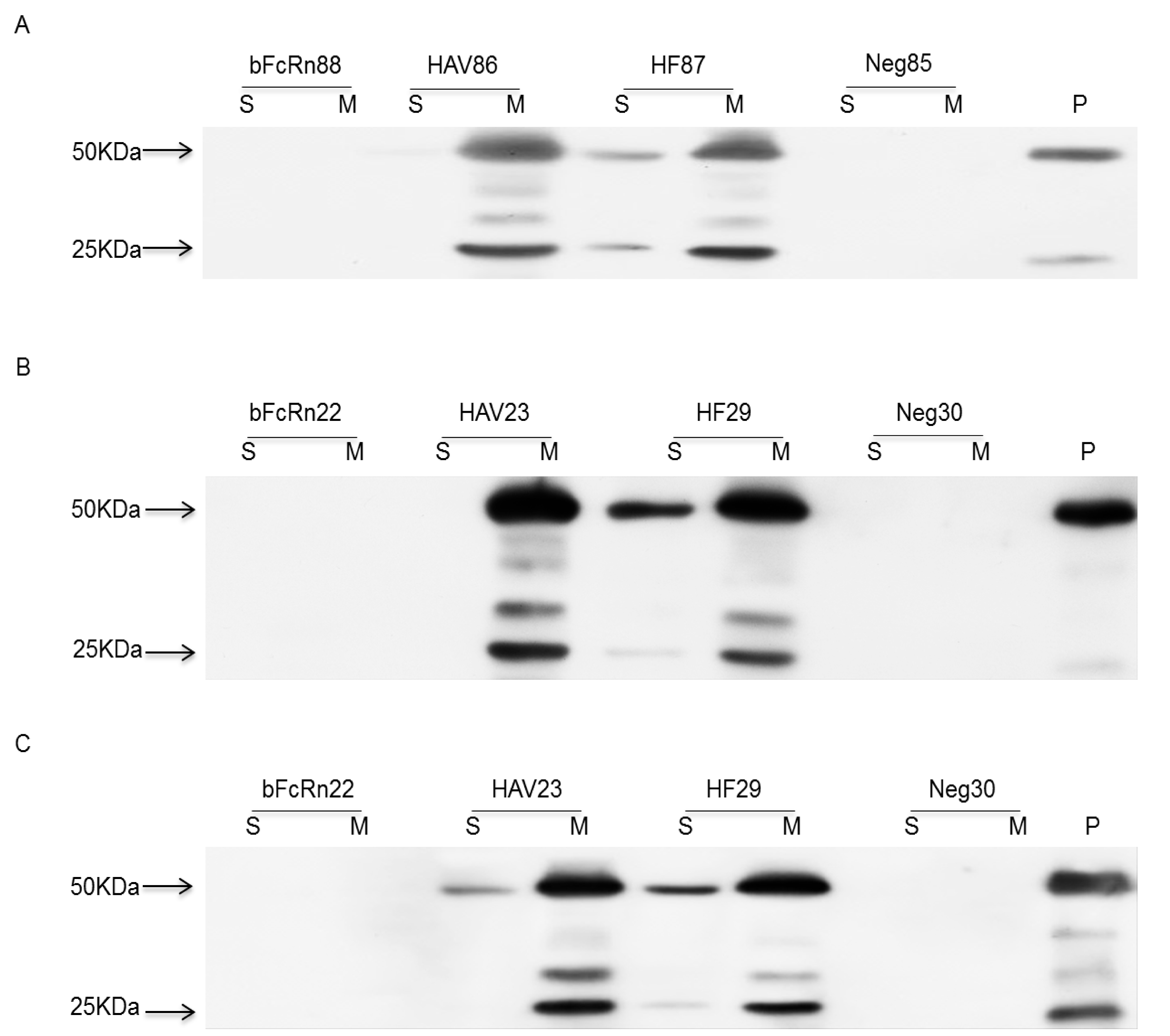

Supplement: S5 Fig — Human IgGs were transferred from milk to serum in the HF transgenic mice. Milk and serum (day 10) in line 1 (S5A) and both day 3 (S5B) and day10 (S5C) in line 42 were performed western blot using goat anti-human IgG antibody in four different genotypes of mice. S, serum; M, milk; P, human IgG. (TIF) [file pone.0115972.s005.tif]

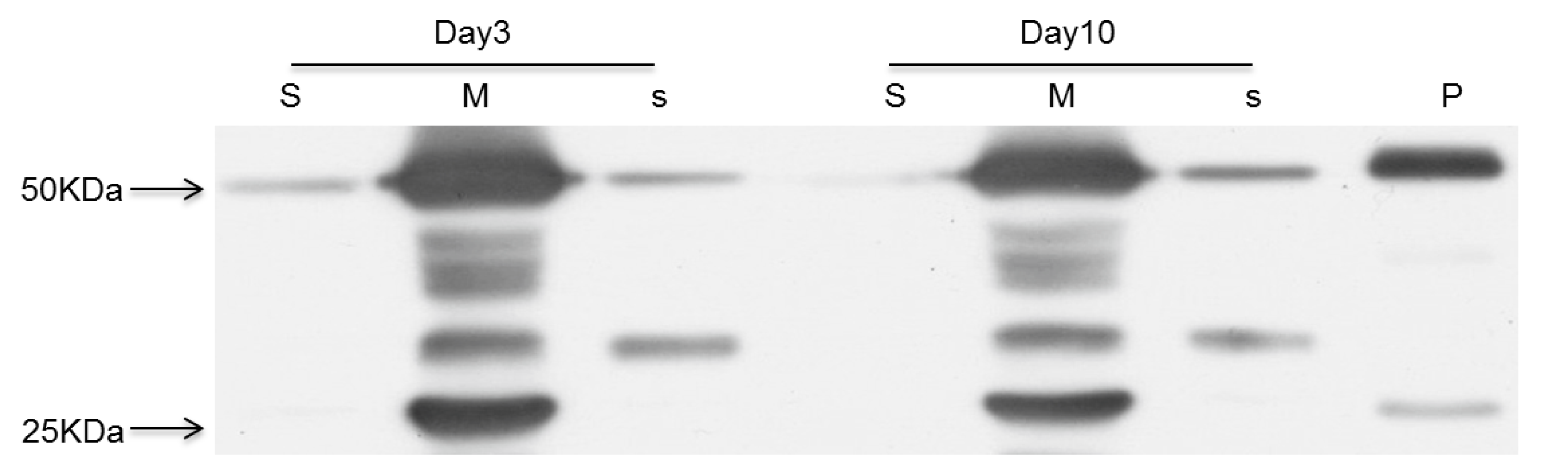

Supplement: S6 Fig — Maternal-fetal human IgGs transfer through suckling. Day3, Day10, two time points were chose to collect milk and serum samples; S, M, s, maternal serum, maternal milk, serum of the neonatal. After the uptake of human IgGs by the neonatal in the intestine, the molecular weight of human IgG LC is larger than that in anti-HAV mAb transgenic mice. (TIF) [file pone.0115972.s006.tif]
